# Supplementary material for: What Does a KAP Survey Reveal about the Awareness Regarding Leishmaniasis among the Community of an Endemic Area in Sri Lanka?
Source: Trop Med Infect Dis. 2024 Feb 28;9(3):55. doi: 10.3390/tropicalmed9030055 (PMC10975155; doi:10.3390/tropicalmed9030055)
Supplement: Supplementary file 1 [file tropicalmed-09-00055-s001.zip › tropicalmed-2657659-Supplementary.pdf]

item no.	Item description
**1**	Do you know about the Leishmaniasis Disease?
**2**	Do you have any idea about the symptoms of the Leishmaniasis disease?
**2a**	Symptom 1 - Wounds
**2b**	Symptom 2 - Blister of pus
**2c**	Symptom 3 - Itching
**2d**	Symptom 4 - Indelible Scars
**2e**	Symptom 5 - Eczema
**2f**	Symptom 6 - All above
**2g**	Symptom 7 - Other
**3**	Do you have any idea about the Leishmaniasis disease?
**4**	Who is the vector of the disease?
**5**	Do you know the control measures of the vectors transmitting leishmaniasis disease?
**6**	Is sandfly only bite in humans?
**7**	Do you have any idea about other biting animals?
**7a**	Domestic Animals
**7b**	Stray Animals
**7c**	Wild Animals
**7d**	Birds
**7e**	Other
8	Do you have an idea about breeding places?
8a	Cultivated Lands
8b	Non-Plastered houses
8c	Animal Pens
8d	Other
8e	All above
10	According to your knowledge, which part of her body does this animal bite?
10a	Do you know the biting Period?
10b	Morning
10c	in the afternoon
10d	Evening
10e	Night
10f	All above
11	Do you know the control measures?
11a	Destroy Breeding Places
11b	Clean/Remove Breeding Places
11c	Check Everyday
11d	Aware of the vector
11e	Other
11f	All above
12	Do you/your family members follow these methods?
13	Do your community members follow these methods?
14	What are the problems do you have to face when reducing the breeding place?

15	What are the steps to be taken in relation to someone suffering from this disease?
16	Do you/your family members follow these methods?
17	Do your community members follow these methods?
18	Do you have an idea about knowledge dissemination?
**19**	Government and Private agencies
**20**	Media
21	Community
22	Are you satisfied with the knowledge of you about the disease and the vector?
23	Are you satisfied with the knowledge of your Family members about the disease and vector?
24	Government Agencies
25	Private Agencies
26	Community Members
27	Family Members
28	You
**29**	All above
**30**	Other
